# Invasive Streptococcus Agalactiae Causing Meningitis, Ventriculitis, and Endocarditis in a Non-Pregnant Adult

**DOI:** 10.7759/cureus.11412

**Published:** 2020-11-10

**Authors:** Amr Abdelradi, Andrew Murphy, Amy M Ahasic

**Affiliations:** 1 Department of Internal Medicine, Norwalk Hospital, Nuvance Health, Norwalk, USA; 2 Department of Pulmonary and Critical Care Medicine, Norwalk Hospital, Nuvance Health, Norwalk, USA; 3 Department of Medicine, Yale School of Medicine, New Haven, USA; 4 Department of Medicine, University of Vermont Larner School of Medicine, Burlington, USA

**Keywords:** group b streptococcus, streptococcus agalactiae, meningitis, ventriculitis, endocarditis

## Abstract

*Streptococcus agalactiae* is a common bacteria known to cause meningitis and urinary tract infections in neonates and pregnant women, respectively. Recently, *S. agalactiae* has become an increasingly recognized pathogen in non-pregnant adults, manifesting most commonly as skin and soft tissue infections, urinary tract infections (UTIs), and pneumonia. Meningitis and endocarditis are among the most feared complications of *S. agalactiae* due to high morbidity and mortality, especially in adults over 65 years of age. Both of these complications are rare. We present a case of simultaneous *S. agalactiae* meningitis and endocarditis in a 69-year-old woman with a history of uncontrolled Type 2 diabetes mellitus. This case emphasizes the importance of prompt recognition and treatment of a complicated invasive *S. agalactiae* infection.

## Introduction

*Streptococcus agalactiae*, also known as Group B *Streptococcus* (GBS), is a well-established cause of infection in neonates and pregnant women where it most commonly manifests as meningitis or urinary tract infections (UTIs), respectively. It is a less common pathogen in non-pregnant adults, with an incidence of approximately 11 per 100,000 patients [1]. Invasive *S. agalactiae* in non-pregnant adults can present as meningitis, endocarditis, and even pneumonia, although these manifestations are rare. In a population-based surveillance study, only 1% of meningitis cases were attributed to GBS in non-pregnant adults, and about 9% of patients with invasive GBS had endocarditis [2]. Prompt recognition and treatment of these complications are important, given the mortality rates of up to 56% and 40% for GBS meningitis and endocarditis, respectively [2-3]. 

## Case presentation

A 69-year-old woman with a history of type 2 diabetes mellitus (DM) and a distant history of total abdominal hysterectomy with bilateral salpingo-oophorectomy due to ovarian cancer presented with progressively worsening confusion, low-grade fevers, nausea, and vomiting for five days prior to admission. She was reportedly compliant with a diabetic diet. Vital signs were notable for a blood pressure of 73/40, heart rate in the 140s in atrial fibrillation, a respiratory rate in the 20s, and a temperature of 34.8 degrees Celsius. On physical examination, she was lethargic but arousable and had no nuchal rigidity. Heart sounds were irregularly irregular, and a Kussmaul breathing pattern was noted. Her abdomen was soft and non-tender. Laboratory workup revealed a blood glucose of 823 mg/dL and a beta-hydroxybutyrate of 12.3 mmol/L. White blood cell count was 17.6 x 109/L with bandemia of 3%. Arterial blood gas was significant for a pH of 6.95, carbon dioxide (PaCO_2_) of 9.9 mmHg, and bicarbonate of 5.8 mmol/L, consistent with severe metabolic acidosis with incomplete respiratory compensation. She was treated for diabetic ketoacidosis (DKA) with intravenous insulin and fluid resuscitation. She was also started on empiric ceftriaxone and vancomycin because of severe sepsis.

**Figure 1:**
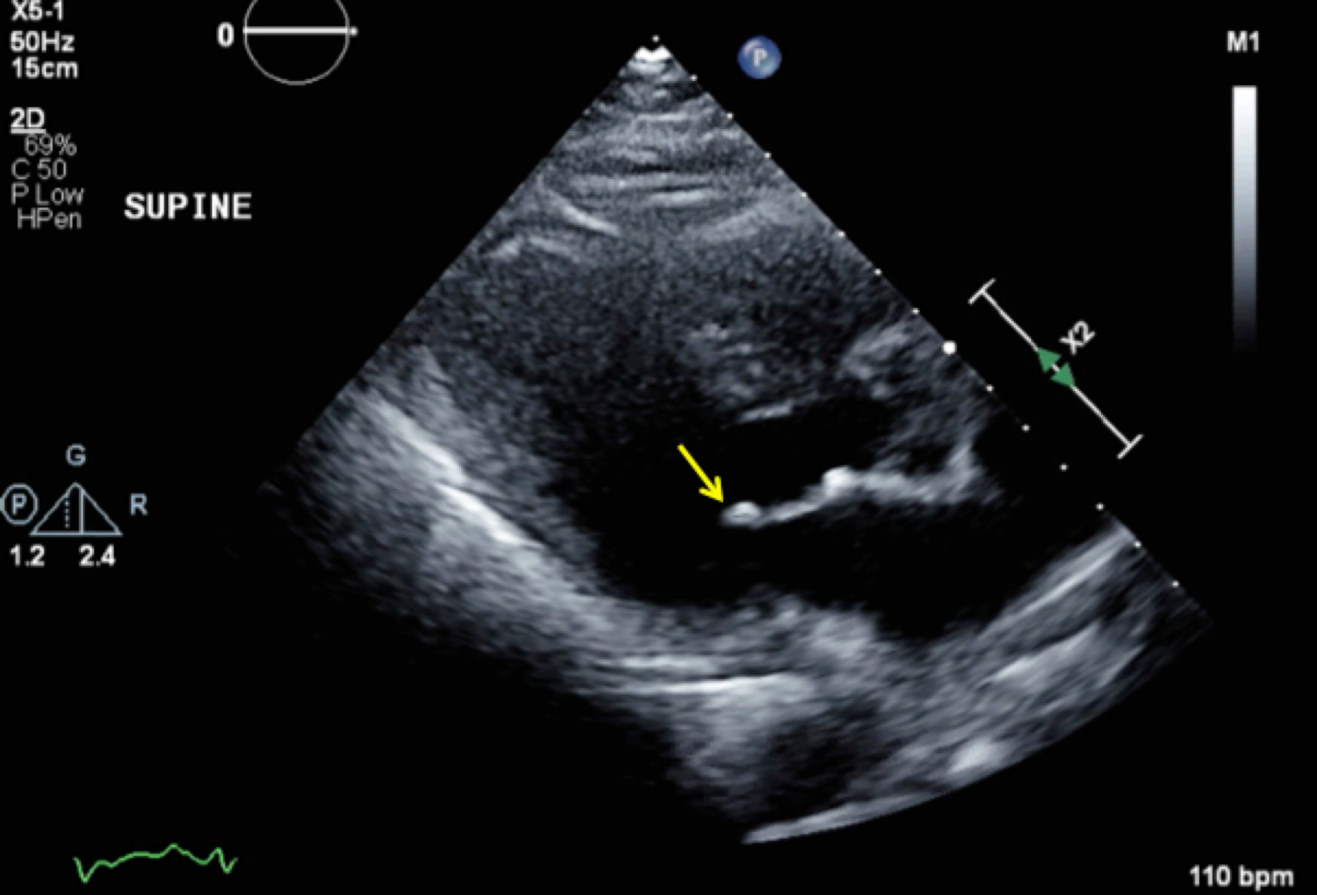
Parasternal long-axis view of the transthoracic echocardiogram (TTE) showing a 0.5 cm vegetation in the anterior leaflet of the mitral valve (yellow arrow)

On the second day of hospitalization, the patient became increasingly lethargic and was minimally responsive. Laboratory workup at the time was significant for leukocytosis of 28 x 109/L and bandemia of 38%. Blood cultures from admission grew *S. agalactiae*. A lumbar puncture was performed, and analysis of the cerebrospinal fluid (CSF) showed a white blood cell count of 4,500/mm^3^, a protein of 354 mg/dL, and a glucose level of 140 mg/dL. Gram stain showed no organisms. Antibiotics were switched to penicillin, plus gentamicin. A transthoracic echocardiogram revealed a 0.5 cm vegetation on the anterior leaflet of the mitral valve with mild mitral regurgitation (Figure 1). A repeat echocardiogram two days later showed no progression in the vegetation size or in valvular dysfunction.

**Figure 2:**
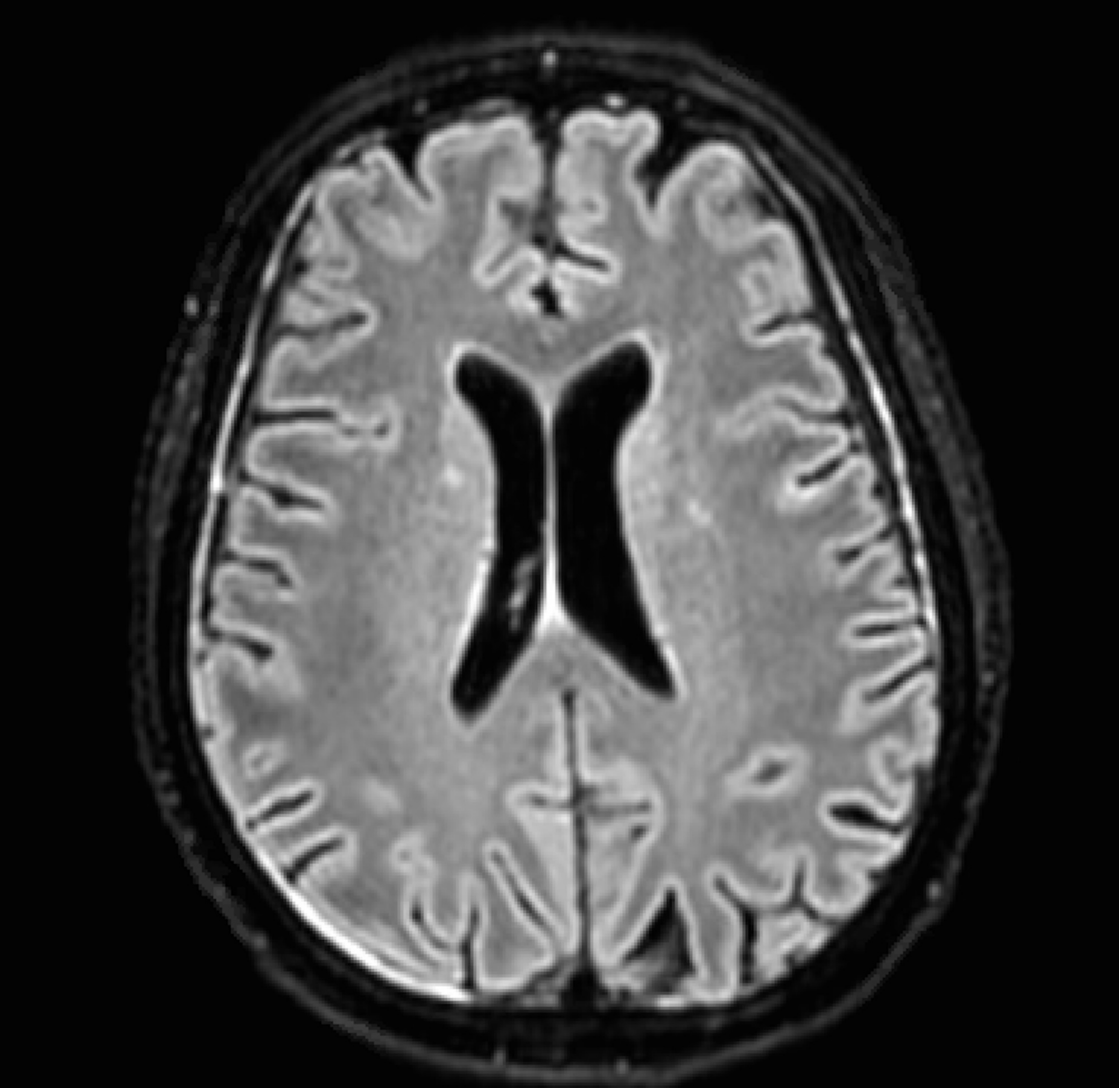
An axial FLAIR MRI shows diffuse, scattered foci of hyperintensity in the bilateral sulci, suggestive of inflammation FLAIR: fluid-attenuated inversion recovery; MRI: magnetic resonance imaging

Her hospital course was further complicated by seizures which were treated with levetiracetam. Magnetic resonance imaging (MRI) of the brain showed the scattered foci of fluid-attenuated inversion recovery (FLAIR) hyperintensity within the bilateral cerebral sulci and ependyma suggestive of meningitis and ventriculitis (Figure 2). Over the next few days, the patient’s mental status improved back to baseline. Her course was also complicated by melena and acute blood loss anemia. Workup revealed a healing, non-bleeding duodenal ulcer. The patient received an initial two weeks of penicillin G, plus gentamicin, followed by an additional two weeks of penicillin G alone. Repeat blood cultures showed no further growth of the GBS. She was discharged to a rehabilitation facility after 30 days of hospitalization.

## Discussion

*S. agalactiae* is gram-positive bacteria that is an uncommon cause of invasive infections in non-pregnant adults. Risk factors for invasive infection include uncontrolled DM, obesity, and malignancy. At the initial presentation, our patient’s altered mental status was attributed to DKA. An infectious process was suspected as the trigger of DKA given her hypothermia and leukocytosis, and as such, she was started on empiric broad-spectrum antibiotics at admission. Genitourinary and pulmonary sources appeared less likely given normal urinalysis and chest X-ray, respectively. Skin infections were also less likely as the patient had no open wounds or skin lesions. Gastrointestinal and central nervous system (CNS) sources could not be immediately ruled out. Given her worsening mental status, leukocytosis, and bandemia despite antibiotic therapy, suspicion for a CNS source of infection increased. A lumbar puncture was therefore performed, and the patient was immediately started on penicillin and gentamicin for GBS meningitis when results of the blood cultures were reported.

Although meningitis and endocarditis are rare manifestations of invasive GBS, the morbidity and mortality can be high. About 4% of adult patients with invasive GBS develop meningitis, which is associated with a mortality rate of 56% in patients above 65 years of age [2, 4]. Mortality is also higher in patients with comorbidities, such as DM. Patients with GBS meningitis have indistinguishable symptomology and exam findings from other causes of bacterial meningitis and will commonly present with altered mental status and somnolence. Ventriculitis, an inflammation of the cerebral ventricular drainage system, is an uncommon complication of community-acquired bacterial meningitis. Most cases of ventriculitis are associated with the presence of indwelling central nervous system devices such as shunts and drains. A report by Gronthoud, et al. identified only seven cases of primary bacterial ventriculitis in the English literature which were caused by various organisms including *Streptococcus aureus*, *Escherichia coli*, and *Listeria monocytogenes* [5]. To our knowledge, no cases of S. agalactiae ventriculitis have been reported. Our patient’s brain MRI showed scattered areas of hyperintensity within the bilateral sulci, as well as ependymal hyperintensity - findings that are consistent with meningitis and ventriculitis.

When meningitis is suspected, prompt lumbar puncture (LP) is warranted for definitive diagnosis. LP is ideally performed prior to antibiotic administration so as to increase test sensitivity. However, antibiotic therapy should not be significantly delayed to perform an LP, which can also be done after initiating treatment, albeit with lower sensitivity. CSF analysis typically reveals elevated protein, leukocytosis, and low glucose. Our patient had hyperglycorrachia, likely due to her concurrent severe hyperglycemia and DKA at presentation. Her CSF culture did not yield bacteria, which can occur in 11% to 30% of meningitis patients [6]. However, the LP was performed one day into the hospital admission after antibiotics had already been initiated, which may have contributed to the negative CSF cultures. CSF sterilization can occur in as little as six hours after antibiotic administration [7].

Endocarditis is another serious complication of invasive GBS that occurs in 2% to 9% of patients with a mortality rate of up to 40% [2-3]. Our patient had evidence of small mitral valve vegetation. Given the patient’s hemodynamic stability and unchanged vegetation size on repeat transthoracic echocardiogram, no surgical intervention was required.

Penicillin is the mainstay treatment for invasive GBS infection. However, third-generation cephalosporins are an acceptable alternative, especially in patients allergic to penicillin. Patients with meningitis or endocarditis may also benefit from the addition of an aminoglycoside for its synergistic effects. The typical duration of treatment depends on the organs involved and the complications of GBS bacteremia. In our patient, the presence of endocarditis necessitated prolonged therapy. Seizures occur in approximately 17% of meningitis patients and are associated with significantly higher mortality than in patients who do not develop seizures (41% vs 16%) [8].

## Conclusions

Invasive GBS infection in non-pregnant adults is becoming an increasingly recognized entity. Serious manifestations, such as meningitis and endocarditis, are uncommon, but they require prompt recognition and treatment as they are associated with significant morbidity and mortality, particularly in older adults. Meningitis should be considered in patients ill with a GBS infection with a low threshold to perform an LP. The current case shows that serious and rare manifestations of disease can coincide. In our patient, meningitis, ventriculitis, and endocarditis all occurred due to GBS.
